# Urinary function, sexual function and quality of life after prostate low-dose-rate brachytherapy: a retrospective analysis

**DOI:** 10.1186/s12894-025-01718-6

**Published:** 2025-02-28

**Authors:** Lauri Mäkelä, Arto Mikkola, Anssi Pétas, Harri Visapää

**Affiliations:** 1 Comprehensive Cancer Center, University of Helsinki and Helsinki University Hospital, Helsinki, Finland; 2 Department of Urology, University of Helsinki and Helsinki University Hospital, Helsinki, Finland

**Keywords:** Prostate cancer, low-dose-rate brachytherapy, Quality of life

## Abstract

**Background:**

Prostate cancer is most commonly diagnosed at a localized stage, allowing the majority of patients to receive curative treatment. The prognosis is often favorable, and there are numerous treatment options available, emphasizing the importance of assessing the side effects associated with each treatment. Low-dose-rate (LDR) brachytherapy is one such treatment option, supported by robust evidence regarding its efficacy and side effects. However, most published data primarily rely on physician-assessed toxicity, which may underestimate the patient's experience of side effects. This study aims to provide a comprehensive overview of the urinary side effects of LDR brachytherapy, with a focus on patient-reported outcomes.

**Methods:**

This retrospective study included 199 patients treated with LDR-brachytherapy years 2000-2012 at Helsinki University Hospital. Questionnaires used to assess urinary toxicity were International Prostate Symptom Score (IPSS) and The Danish Prostatic Symptom Score (DAN-PSS). Additionally, sexual function was assessed using the International Index of Erectile Function (IIEF) questionnaire. Urinary function was assessed with flowmetry, reporting peak flow rate and postvoid residual measurement.

**Results:**

There was a deterioration in urinary function during the first six months post-procedure, as evidenced by a decline in the urinary function questionnaires and uroflowmetry measurements. For most patients, this deterioration was transient, with median symptom scores returning to baseline after one year. A slight discrepancy was observed between patient-reported outcome measures and urinary function assessed by flowmetry.

**Conclusions:**

LDR brachytherapy is a well-tolerated treatment for localized prostate cancer. While many patients experience acute side effects that subside relatively quickly, there is a small risk of prolonged side effects. This risk should be thoroughly discussed with patients when making treatment decisions.

## Background

Most cancer treatments carry adverse effects that significantly impact patients' quality of life. The careful estimation of the risks and benefits associated with each treatment option is especially important for cancer patients who are being offered curative treatment. This is especially relevant for prostate cancer, as approximately 83% of diagnoses are made at the localized stage [1]. Recently, ProtecT-trial reported 15-year outcomes after monitoring, surgery, or radiotherapy for prostate cancer. Of 1610 patients whose follow-up was complete, death from prostate cancer occurred only in 45 (2.7%) men [2]. This further highlights the importance of evaluating how each treatment choice impacts patients' daily lives, particularly in low- and intermediate-risk prostate cancer patients for whom active surveillance could be a viable option [3].

LDR-brachytherapy is one of the many options available for treating low-, and intermediate-risk localized prostate cancer [4]. There is robust evidence on efficacy and tolerability of the treatment from previous studies [5–12]. However, a challenge arises when interpreting the results concerning treatment tolerance. It has been shown that physician- and patient reported assessments of health-related quality of life do not completely correlate [13, 14]. Therefore, studies reporting patient reported outcomes offer relevant information for doctors and patients when different treatment options are discussed. We hypothesized that the favorable tolerability of LDR-brachytherapy observed in previous studies would also be reflected in patient-reported outcomes and functional measurements among patients treated at our institution.

## Methods and materials

One hundred ninety-nine patients treated with LDR-brachytherapy using Iodine-125 seeds during years 2000–2012 at Helsinki University Hospital were included in the study. The patient selection criteria were a prostate volume of <50 cm^3^ and a Gleason score of <7. However, these criteria were not strictly enforced, as some patients who did not meet them were still treated. There was no predetermined threshold for excluding patients based on questionnaire scores. Questionnaires were collected and urinary function assessed before the treatment and during the follow-up visits. Follow-up visits were arranged at 1, 3 and 6 months and with six-month intervals thereafter for up to 5 years.

Questionnaires used to assess urinary toxicity were International Prostate Symptom Score (IPSS) and The Danish Prostatic Symptom Score (DAN-PSS) [15, 16]. In both questionnaires, a score of 7 is considered mildly symptomatic, while scores of 8–19 indicate moderate symptoms, and scores of 20–35 indicate severe symptoms. Additionally, the IPSS questionnaire included a 7-point scale question asking the patient how they would feel if they were to spend the rest of their life with their current urinary condition. This was recorded separately and presented in this study as a quality of life (QOL) score. The scores ranged from 0 to 6, with higher scores indicating a relatively lower quality of life.

Sexual function was assessed using the International Index of Erectile Function (IIEF) questionnaire, with the total score (possible range 0–75) recorded. This questionnaire comprises five factors: erectile function, orgasmic function, sexual desire, intercourse satisfaction, and overall satisfaction [17].

Urinary function was assessed with flowmetry reporting peak flow rate (ml/s) and postvoid residual measurement.

### Statistical analysis

Descriptive statistics were used to assess changes in questionnaire scores over time and simple error bar means with 95% confidence intervals were presented as graphs. A one-sample T-test was performed to assess the change in symptom scores at different timepoints compared to the mean of pre-treatment symptom scores. To counteract the multiple comparisons problem, the Bonferroni correction method was used. Thus, *p*-value of 0.004 (0.05/12) was used to determine the statistical significance in these comparisons.

For a closer analysis of IPSS scores and flowmetry, a baseline values and a follow-up of at least 12 months with minimum of two recorded timepoints was required. IPSS resolution was defined as a return of the IPSS score to within 2 points of the baseline score. Furthermore, patients who had follow-up measurements at 18 or 24 months were separately analyzed to identify patients who then had a second worsening in at ≥1 year. In IPSS this was defined as an increase of ≥5 points from the nadir and in a uroflowmetry a decline of at least 5 ml/s in the peak flowrate from the highest flowrate measured after brachytherapy.

## Results

A total of 199 patients were included in the study. The median follow-up time was 36 months (IQR 12–54). The median age of the patients was 62 years (IQR 57–66). Detailed patient characteristics are presented in Table 1. At baseline, most patients presented with low urinary symptom scores and good urinary function. However, 5 patients had a baseline IPSS score over 20, indicating severe urinary symptoms. 13 patients presented with a flowrate under 10ml/s. 11 patients had residual urine greater than 100ml and there was a correlation between larger prostate size and urinary residual (Pearson correlation= .269 *p*=0.021). Adjuvant hormonal treatment was rarely used as these were low-, and intermediate risk patients. Median baseline IIEF total score was 57.5 (IQR 28–64) implying a reasonably good sexual health at baseline.

**Table 1:** Patient characteristics

Patient Characteristics (*n*=199)	
Age median (range)	62 (46–82)
Primary Gleason score	
missing	2
≤6	176
7 (3+4)	19
7 (4+3)	2
Prostate volume median (range)	34.5 cm3 (15.1–61.6cm3)
Primary PSA median (range)	6.1 (1.7–20.4)
ADT	
yes	4
no	195
Treatment information	
No of needles median (range)	24 (16–34)
No of seeds median (range)	73 (43–105)
Prostate D90 (Gy) median *n*=74	177 (149–195)
Baseline Questionnaire scores	
Dan-PSS median (range) *n*=94	4 (0–71)
IPSS median (range) *n*=105	5 (0–35)
QOL median (range) *n*=89	1 (0–5)
IIEF median (range) *n*=82	57.5 (0–74)
Baseline urinary function	
Urinary peak flow rate (ml/s) median (range) *n*=90	17.45 (3.6–33.3)
Post-void residual urine (ml) median (range) *n*=74	29 (0–332)

**Fig. 1:**
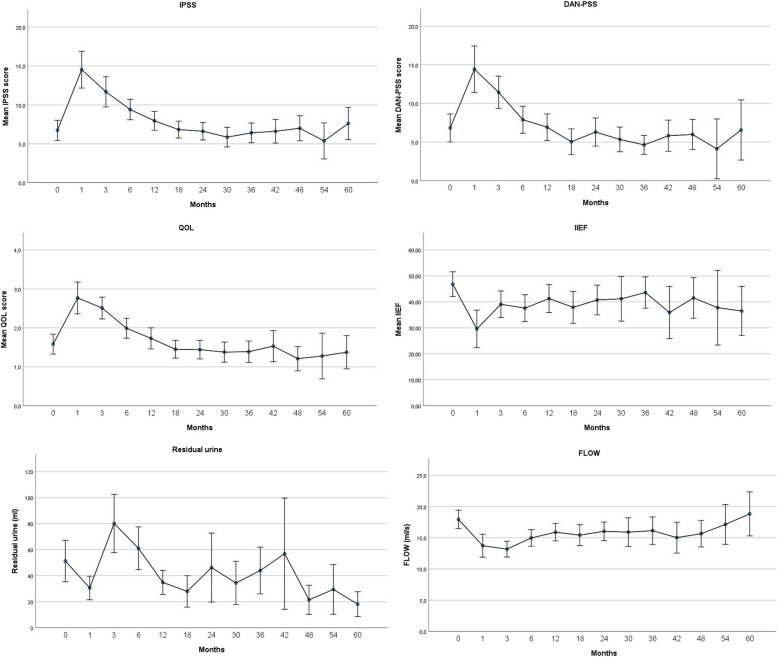
Chronological changes in symptom scores, flowmetry, and post-void residual measurements from baseline

The chronological changes from the baseline in symptom scores, flowmetry and post-void residual measurements are shown in Fig. 1. There was a statistically significant (*p*<0.004) worsening in IPSS, QOL, IIEF scores at 1,3 and 6 months. In DAN-PSS score change was statistically significant at 1 and 3 months. A resolution of patient-reported urinary symptoms and quality-of-life could be seen after 6 months as these scores returned to baseline at 12 months. In the flowmetry, the change from the baseline was statistically significant in each of the timepoints up until 18 months. Compared to other parameters, changes in the post-void residual volume were more inconsistent and changes from the baseline were statistically significant only at 1, 12 and 18 months.

Tested with one-sample T-test, IPSS and DAN-PSS symptom scores correlated with each other at each timepoint. Further analysis of urinary symptoms was performed using the IPSS questionnaire and flowmetry measurements.

### IPSS

Ninety-five patients had an adequate amount of IPSS scores at different timepoints and were included in closer analysis of the IPSS resolution. Median follow-up time in this cohort was 36 months (IQR 24–54). Mean time to IPSS resolution was 7.65 months. 26 patients (27.4%) did not have an IPSS resolution at 12 months. 14 patients (14.7%) did not experience an IPSS resolution at any point during the follow-up. 79 patients were eligible for analysis of a urinary symptom flare. This was evidenced in 12 patients (12.6%) and occurred at 12–24 months.

### Flowmetry

Eighty patients had an adequate amount of flowmetry data at different timepoints and were included in closer analysis. Median follow-up time in this cohort was 42 months (IQR 36–57). In 44 patients (55%) urinary peak flow rate returned to baseline during the follow-up. The mean time for the flow rate to return to baseline was 16 months, with only 20 patients (25%) achieving a flow rate at baseline or higher at 12 months. The mean time for the flow rate to return to baseline was 16 months. 55 patients were eligible for analysis of symptom flare. After an initial recovery of flow rate at 6–12 months, a second decline was observed in 20 patients (37.7%) at 12–24 months. One of these patients required surgical intervention due to urethral stricture, which led to a decline in urinary flow rate and subsequent voiding problems. The patient underwent an optical urethrotomy after 18 months of follow-up, as his flow rate decreased to 3.2 ml/s.

## Discussion

In this study, we assessed a wide range of patient-reported outcomes as well as functional measurements after LDR-brachytherapy. We observed a clear deterioration in urinary function during the first 6 months post-procedure. For most patients, this deterioration was transient, as median symptom scores returned to baseline after one year. These scores remained relatively stable thereafter during the later stages of the 5-year follow-up, implying good long-term tolerability of the treatment. These findings are consistent with previous studies, where IPSS scores were reported to return to baseline levels in approximately 85% of patients [18–20].

A slight discrepancy was observed between the patient-reported outcome measures and urinary function assessed by flowmetry. While flow rate remained below baseline for almost half of the patients, this did not significantly impact patient-reported quality of life, as these outcomes returned to baseline earlier. Post-void residual urine results appeared to align with urinary symptom scores and flowmetry results, but there was significantly more deviation in the results across different time points.

A transient symptom flare of lower urinary tract symptoms occurring at approximately 2 years after brachytherapy has been described previously [21, 22]. In our patient cohort, this was most notable in flowmetry measurements, with over a third of the patients experiencing a transient decline in flow rate. Still, this occurrence was less frequent than expected as Keyes et. al reported a urinary symptom flare in 52 percent of patients after LDR-brachytherapy [22]. This discrepancy could be attributed to differences in brachytherapy techniques as well as the patient population.

Only 4 patients had adjuvant hormonal treatment combined to the treatment allowing for clearer observation of changes in sexual function scores, primarily reflecting the impact of brachytherapy alone. Like the urinary symptoms, there was an initial deterioration and gradual improvement in sexual function. While it's been shown that erectile dysfunction following LDR-brachytherapy typically develops gradually and is often permanent, our study suggests that the most significant impact on overall sexual health occurred at the 6 months with a gradual improvement thereafter [23].

Since the first patients in this study were treated over 20 years ago, it is important to acknowledge that treatment practices have evolved. Most patients in this cohort were low risk, with a Gleason score of 6, for whom active surveillance would now be the recommended approach [24]. This is also the current practice at our institution. However, as this study focuses on functional outcomes and quality of life, we believe that changes in treatment practices do not diminish the relevance of our findings.

There are several limitations to this study. We only collected total scores of the questionnaires, so we were unable to provide a breakdown of different subsections. Therefore, the exact nature of the urinary problems remained unclear; for instance, whether the symptoms were more irritative or obstructive, or if there was any incontinence. The same applies to sexual function, where only the IIEF-5 total score was recorded, encompassing a wide variety of factors affecting sexual function. Besides hormonal therapy, we were not able to gather data on patients' medication at the time of brachytherapy or during the follow-up. For example, the use of alpha blockers could affect urinary symptoms and flow rate, limiting the interpretability of these findings. Furthermore, this study is limited by being a single-center study. As brachytherapy is an operator-sensitive treatment modality, there could be differences in outcomes between different centers.

## Conclusions

We have provided a comprehensive overview of various domains of patient-reported outcomes and urinary function following prostate LDR-brachytherapy. Our findings suggest that patient-reported urinary side effects are largely transient in nature, with most patients not experiencing substantial long-term side effects. However, a minority of patients do experience longer-term side effects, which may significantly impact their daily lives. Therefore, it is imperative that these risks are thoroughly discussed with patients, especially when treating malignancies with a favorable prognosis, such as low- and intermediate-risk prostate cancer. Prospective studies focusing on patient-reported outcomes, factors influencing them, and comparisons with different treatment modalities are warranted.

## Data Availability

The datasets used and/or analysed during the current study are available from the corresponding author on reasonable request.
